# A retrospective examination of care pathways in individuals with treatment-resistant depression

**DOI:** 10.1192/bjo.2021.59

**Published:** 2021-05-14

**Authors:** Elana Day, Rupal Shah, Rachael W. Taylor, Lindsey Marwood, Kimberley Nortey, Jade Harvey, R. Hamish McAllister-Williams, John R. Geddes, Alvaro Barrera, Allan H. Young, Anthony J. Cleare, Rebecca Strawbridge

**Affiliations:** Department of Psychological Medicine, Institute of Psychiatry, Psychology & Neuroscience, King's College London, UK; and National Affective Disorders Service, South London & Maudsley NHS Foundation Trust, UK; Department of Psychological Medicine, Institute of Psychiatry, Psychology & Neuroscience, King's College London, UK; and National Affective Disorders Service, South London & Maudsley NHS Foundation Trust, UK; Department of Psychological Medicine, Institute of Psychiatry, Psychology & Neuroscience, King's College London, UK; Department of Psychological Medicine, Institute of Psychiatry, Psychology & Neuroscience, King's College London, UK; Academic Psychiatry, Cumbria, Northumberland, Tyne and Wear NHS Foundation Trust, UK; and Northern Centre for Mood Disorders, Translational and Clinical Research Institute, Newcastle University, UK; Research Delivery, Oxford Health NHS Foundation Trust, UK; Academic Psychiatry, Cumbria, Northumberland, Tyne and Wear NHS Foundation Trust, UK; and Northern Centre for Mood Disorders, Translational and Clinical Research Institute, Newcastle University, UK; Research Delivery, Oxford Health NHS Foundation Trust, UK; and Department of Psychiatry, Oxford University, UK; Research Delivery, Oxford Health NHS Foundation Trust, UK; and Department of Psychiatry, Oxford University, UK; Department of Psychological Medicine, Institute of Psychiatry, Psychology & Neuroscience, King's College London, UK; and National Affective Disorders Service, South London & Maudsley NHS Foundation Trust, UK; Department of Psychological Medicine, Institute of Psychiatry, Psychology & Neuroscience, King's College London, UK; and National Affective Disorders Service, South London & Maudsley NHS Foundation Trust, UK; Department of Psychological Medicine, Institute of Psychiatry, Psychology & Neuroscience, King's College London, UK

**Keywords:** Depression, depressive disorders, treatment-resistant depression, stepped care, clinical guidelines

## Abstract

**Background:**

Individuals with treatment-resistant depression (TRD) experience a high burden of illness. Current guidelines recommend a stepped care approach for treating depression, but the extent to which best-practice care pathways are adhered to is unclear.

**Aims:**

To explore the extent and nature of ‘treatment gaps’ (non-adherence to stepped care pathways) experienced by a sample of patients with established TRD (non-response to two or more adequate treatments in the current depressive episode) across three cities in the UK.

**Method:**

Five treatment gaps were considered and compared with guidelines, in a cross-sectional retrospective analysis: delay to receiving treatment, lack of access to psychological therapies, delays to medication changes, delays to adjunctive (pharmacological augmentation) treatment and lack of access to secondary care. We additionally explored participant characteristics associated with the extent of treatment gaps experienced.

**Results:**

Of 178 patients with TRD, 47% had been in the current depressive episode for >1 year before initiating antidepressants; 53% had received adequate psychological therapy. A total of 47 and 51% had remained on an unsuccessful first and second antidepressant trial respectively for >16 weeks, and 24 and 27% for >1 year before medication switch, respectively. Further, 54% had tried three or more antidepressant medications within their episode, and only 11% had received adjunctive treatment.

**Conclusions:**

There appears to be a considerable difference between treatment guidelines for depression and the reality of care received by people with TRD. Future research examining representative samples of patients could determine recommendations for optimising care pathways, and ultimately outcomes, for individuals with this illness.

Suboptimal response to treatment for major depressive disorder (MDD) is common. Only around a third of depressed individuals fully respond to their first treatment, around two-thirds only experience a partial response to multiple antidepressant treatments, and approximately 15–30% experience established treatment-resistant depression (TRD), defined as an insufficient response to two or more antidepressant treatments within an episode.^1,2^ Overall, psychological and pharmacological treatments appear to be equally effective in producing a clinical response.^3^ Therefore, many patients with depression may receive a series of ineffective treatments for their condition, prolonging illness, distress and disability. Patients with TRD suffer a disproportionately higher burden of illness, with poorer health-related quality of life, increased mortality, higher relapse rates and resultant economic costs compared with non-TRD,^4^ with the burden increasing in congruence with the severity of treatment resistance.^5^

Currently, the method recommend by the National Institute for Health and Care Excellence (NICE) for treating patients with depression in the UK is the stepped care approach.^6^ Within this model, all patients with potential depression are initially assessed and provided with psychoeducation tools. They should be actively monitored, and those who do not demonstrate an adequate response should be ‘stepped up’ to a more intensive treatment category. Subsequently, everyone with persistent depression (step 2) should be offered low-intensity evidence-based psychological treatment and medication by default, before being offered high-intensity interventions and combination treatment (step 3). Step 4, including in-patient care and electroconvulsive therapy (ECT), is reserved for emergency situations or complex cases following the failure of previous treatment steps (see Fig. 1). Adhering to NICE stepped care guidelines is widely thought to improve outcomes for depression, particularly if patients and healthcare practitioners work together.^7^

**Fig. 1:**
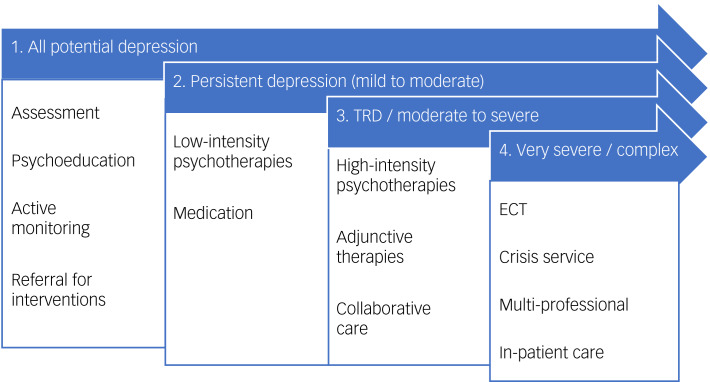
Guideline stepped care treatment pathway. This figure summarises the stepped care pathway for depression, as utilised by the 2009 National Institute for Health and Clinical Excellence guidelines.^6^ Note that this depiction does not capture continuity of care or timelines for progression and management (within and between stages), which are expanded on in treatment guidelines. Collaborative care refers to the multi-component care of a patient, with case managers, primary care clinicians and mental health specialists in communication; this may also incorporate measurement-based care. ECT, electroconvulsive therapy; TRD, treatment-resistant depression.

The extent to which stepped care models are adhered to for people experiencing depression is not well understood and varies between services,^8^ but there is evidence to indicate substantial variation between individuals in treatments, duration, delays and outcomes. We use the term ‘treatment gap’ to refer to a non-adherence to best-practice stepped care pathways.

One barrier to patients receiving effective treatment is the low detection rate of depression in healthcare services. This leads to untreated illness,^9^ and clearly prevents stepped care from being implemented. Those experiencing TRD have, by definition, initiated care pathways, but treatment gaps may still contribute to poor outcomes; for example, through the failure to initiate or adhere to subsequent treatment steps. One study looking at people with early-stage TRD (who had not responded to one adequate treatment in their current depressive episode) suggests that this is the case, and patients with TRD are not being treated according to best-practice guidelines. Wiles et al^10^ found that a high proportion of patients, over a period of 12 months, remained on the same medication at the same dose despite remaining depressed. Antidepressant switching or combination was uncommon, and few patients accessed other treatments or were referred to secondary care. However, adherence to care pathway recommendations has not, to our knowledge, been examined for individuals with established TRD as defined by the consensus-based definition of non-response to two adequate antidepressants within a depressive episode.^11^ Moreover, factors that explain variation in care pathway adherence are not clear.

This work focuses on five treatment gaps, specified below and predefined in line with the NICE stepped care pathway (Fig. 1). These focus on gaps within a depressive episode, rather than across multiple episodes across the lifetime, in line with treatment guidelines. The first treatment gap is ‘time to antidepressant initiation’. Prior evidence suggests that a greater delay to treatment for a depressive episode is a common problem (whether because of detection or stigma-related factors, lack of access to care or other factors^8^) and is associated with reduced response prospectively, and thus may precede development of TRD.^12^ The second treatment gap is ‘access to psychological therapies’. Treatment guidelines recommend a psychological therapy (step 2 onward) and a combination of pharmacological with psychological treatment for those with moderate or severe depression (step 3),^6,11^ as this leads to better clinical outcomes compared with either treatment alone.^13^ The third treatment gap is ‘time to medication changes’. If a person has not responded to an antidepressant treatment after 6–8 weeks, they are unlikely to achieve response in the longer term, and therefore switching to another antidepressant after 4–8 weeks is recommended by guidelines.^6^ This was identified as a treatment gap by Wiles et al.^10^

The fourth treatment gap is ‘steps to adjunctive treatment’. As part of the best-practice stepped care treatment pathways, all the major UK prescribing guidelines recommend augmentation with atypical antipsychotics or lithium when individuals with depression have failed to adequately respond to two first-line treatments,^14^ and this is supported by meta-analysis.^15^ After two unsuccessful monotherapy trials of antidepressants, switching to a third tends to result in reduced response rates,^16^ whereas augmenting increases response rates.^17^ Therefore, being treated with more than two antidepressant monotherapies within the current episode without an adequate response, before adjunctive treatment is implemented (e.g. as specified in the Maudsley Treatment Inventory (MTI)),^18^ would be considered a treatment gap. The fifth treatment gap is ‘access to secondary care’. Treatment guidelines recommend adjunctive treatment prescription and monitoring in secondary care, or in consultation with a secondary care psychiatrist.^6,11^ Patients with more than two antidepressant monotherapy treatment trials in the current episode and/or those to be initiated on an adjunctive treatment should therefore be referred to secondary care. Lack of access to secondary care was highlighted as a treatment gap by Wiles et al.^10^

A sixth predefined outcome was requirement for a step 4 intervention (in-patient admission or ECT), recommended in emergency situations. The use of step 4 care is therefore determined by the clinical presentation of patients, rather than the failure of a preceding step in the care pathway. As such, lack of a step 4 intervention is not considered a treatment gap in this study.

## Objectives

In light of the burden of established TRD and the findings from Wiles et al^10^ indicating poor adherence to recommended pathways in early-stage TRD, our primary objective was to estimate the extent to which an opportunistic sample of individuals with TRD were treated according to current best-practice stepped care guidelines in the UK. Second, we aimed to examine participant characteristics in association with the extent of treatment gaps experienced within care pathways. Data pertaining to the five treatments gap specified above were examined as outcomes.

## Method

### Design

The Lithium versus Quetiapine in Depression (LQD) study is a multicentre, phase 4, randomised clinical trial comparing the clinical and cost effectiveness of lithium versus quetiapine augmentation in patients with TRD.^19^ The study was designed to be as pragmatic as possible, meaning the participant sample and intervention protocol will reflect real-world clinical practice, within the constraints of clinical trial regulations. One element of the trial's pragmatism was the aim of recruiting patients at the point at which a clinician was, or should be, already considering prescribing a pharmacological augmenter, which could include lithium or quetiapine (the trial medications) as recommended first-line augmentation treatments in normal clinical practice.^11,20^

Approval for the LQD study (clinicaltrials.gov: NCT03004521)was obtained from the Cambridge South Research Ethics Committee (16/EE/0318), the Medicines and Healthcare Products Regulatory Authority (EudraCT: 2016–001637-27) and the Health Research Authority. Written informed consent was provided by all participants.

This paper is a cross-sectional subanalysis, using baseline LQD data collected before randomisation and initiation with lithium or quetiapine. This study included participants from three of the study sites: London (South London and Maudsley National Health Service (NHS) Foundation Trust), Oxford (Oxford Health NHS Foundation Trust) and Newcastle (Cumbria, Northumberland, Tyne and Wear NHS Foundation Trust). Participants were recruited through primary and secondary care services, as well as advertisements in the community.

### Participants

As described in the study protocol,^19^ eligible participants were aged 18 years or over, were under the care of a general practitioner or mental health service, and had been taking the same antidepressant medication at an adequate therapeutic dose for at least 6 weeks. Participants were also required to score ≥14 on the 17-item Hamilton Rating Scale for Depression.^21^ representing at least a moderate severity of depression; meet the DSM-5 criteria for MDD on the Mini International Neuropsychiatric Interview 7.0^22^; and meet the criteria for TRD, defined as not responding to at least two antidepressants in the current depressive episode, taken for at least 6 weeks at a minimum therapeutic dose, as defined by the Maudsley Prescribing Guidelines (MPG).^23^

Participants with bipolar disorder or a diagnosis of current psychosis were excluded. Anyone who had taken an adequate dose of lithium or quetiapine in the current episode of depression were also excluded, as were patients with a known contraindication to either medication. All other comorbidities were permitted.

### Measures

#### Treatment gaps

The prespecified treatment gap outcomes were addressed by comparing the following study data points with the NICE^6^ and British Association for Psychopharmacology (BAP)^11^ guidelines for depression, which are summarised in Table 2. For time to antidepressant initiation, the time between date of episode onset and date of first antidepressant treatment within the episode (patient-reported) was computed into categories (already treated, 0–3 months, 4–12 months, 13–24 months and >24 months). To determine access to psychological therapies, we used patient-reported information provided and examined whether participants had undertaken any form/intensity of psychotherapy and undertaken an adequate course (as per NICE guidelines recommendation; see Table 2), as well as the number of therapies completed.

To determine time to medication changes, we used information on the MTI medication form (dates and dosages of each antidepressant taken) to calculate the duration of each of the first two antidepressants taken within the episode (categorised as 0–6 weeks, 7–16 weeks, 17–52 weeks and >52 weeks). Although guidelines recommend remaining on an antidepressant for 4–8 weeks, there is some lack of consensus and titration may be needed to achieve a response;^23^ we conservatively consider ≤16 weeks as putatively not representing a substantial treatment gap. Unsuccessful treatment is defined by a patient not experiencing >2 weeks without depression, delineating their current episode and causing any treatment tried in this period to be unsuccessful by definition. For steps to adjunctive treatment, the number of antidepressant monotherapies taken within the current episode was calculated via the MTI medication form, computing the number of antidepressants of any duration in addition to the number of adequate trials of medication (>6 weeks at an adequate dose according to the MTI). Note that the minimum number of adequate antidepressant trials for inclusion in the study was two. We also assessed whether individuals had taken adjunctive medications within their episode, using the MTI form and medical notes (any dose/duration) and the number of first- and second-line adjunctive treatments as defined by the MTI medication form, in line with the MPG (any dose/duration and for an adequate course, i.e. 6 weeks, as defined above).^18,23^

For access to secondary care, participants noted whether they were under the care of a psychiatrist or secondary care service, reported as a binary variable. To determine requirement for step 4 intervention, we examined whether a participant had undertaken ECT in the current depressive episode (from the Maudsley Staging Method (MSM^18^) and whether a participant had been admitted as an in-patient for depression within the prior 3 months (from the Client Service Receipt Inventory^24^).

#### Demographics and measures of depression burden

The following demographic and clinical characteristics were considered in association with potential treatment gaps: age, identified gender, ethnicity, employment status, relationship status, level of education, recruitment method (community/primary care/secondary care), physical comorbidity, number of current medications (psychotropic and non-psychotropic), number of psychiatric comorbidities, number of past episodes of depression, current depressive episode duration, depression severity (Montgomery–Åsberg Depression Rating Scale (MADRS^25^)), treatment resistance (MSM^18^) and psychosocial functioning (Work and Social Adjustment Scale (WSAS^26^)). With the exception of validated investigator-rated (MSM, MADRS) or participant-rated (WSAS) measures, the aforementioned information collected was through self-report forms, as part of the LQD study screening and baseline visit.

### Data analysis

Variables were tested for normality and for non-normal distributions, non-parametric tests were employed. Continuous variables were described with median and interquartile range. Percentages were calculated for the entire sample (*N* = 178), with missing data included. To investigate the primary objective, an exploratory comparison of the participant sample with best-practice guidelines was conducted, tentatively interpreted as whether each treatment gap outcome indicated a potentially significantly difference from NICE stepped care guidelines based on 95% confidence intervals; where 95% confidence intervals overlap with the guideline recommendation was defined as indicating adherence to guidelines. Overlap of 95% confidence intervals are also used to indicate differences between participants recruited from the different study sites (London, Oxford and Newcastle).

To investigate the secondary objective, treatment gap variables were compared with participant characteristics by using appropriate univariate tests: *χ*^2^-tests were used to compare nominal variables with binary variables, Mann-Whitney *U*-tests were used to compare continuous/ordinal variables with binary variables, ANOVA was used to compare multi-categorical variables with continuous variables and Spearman's correlation was used to compare continuous variables. All analyses were undertaken in SPSSversion 26 for Windows.

## Results

### Participant characteristics

In total, 178 patients (73 from London, 52 from Newcastle, 53 from Oxford) were assessed. Table 1 summarises participant characteristics. The Newcastle site had fewer Black and minority ethnic (BAME) participants (2%) compared with London (19%). A greater proportion of participants from the Oxford site (62%) were in employment compared with London (45%) and Newcastle (35%). Oxford also had a higher proportion of participants in a long-term relationship (53%) than London (38%) and Newcastle (39%).

**Table 1:** Participant characteristics

Variable	*N*	All (*N* = 178)	London (*n* = 73)	Newcastle (*n* = 52)	Oxford (*n* = 53)
		% [95% CI]			
Binary					
Gender					
Female (versus male)	178	55% [47–62%]	56% [44–67%]	54% [41–66%]	53% [40–66%]
Physical comorbidity					
Yes (versus no)	177	82% [76–87%]	86% [76–93%]	83% [70–91%]	74% [62–85%]
Lifetime suicide attempt					
Yes (versus no)	177	39% [33–47%]	41% [31–53%]	48% [35–61%]	28% [19–44%]
Ethnicity					
BAME (versus White)^a^	177	11% [7–16%]	19% [12–30%]	2% [0–11%]	8% [3–19%]
Employed					
Yes (versus no)^b^	178	47% [40–55%]	45% [34–57%]	35% [23–48%]	62% [49–74%]
Long-term relationship					
Yes (versus no)	177	43% [36–50%]	38% [28–50%]	39% [26–52%]	53% [40–66%]
Multi-categorical					
Education					
Primary/less	7	4% [2–8%]	6% [2–14%]	2% [0–11%]	4% [0–14%]
Secondary	25	14% [10–20%]	16% [10–27%]	14% [6–26%]	11% [5–24%]
College (further)	72	40% [34–48%]	36% [26–47%]	52% [39–65%]	36% [24–49%]
Degree (higher)	48	27% [21–34%]	32% [22–43%]	21% [12–34%]	26% [17–41%]
Postgraduate	26	15% [10–21%]	11% [5–20%]	12% [5–23%]	23% [13–36%]
Recruitment method					
Community^c^	38	21% [16–28%]	44% [34–56%]	6% [1–16%]	6% [1–16%]
Primary care^d^	52	29% [23–36%]	43% [32–55%]	33% [21–47%]	8% [2–18%]
Secondary care^e^	87	49% [42–56%]	12% [7–22%]	66% [48–74%]	87% [75–94%]
Number of current medications^f^					
0	31	17% [13–24%]	19% [12–30%]	12% [5–23%]	21% [12–34%]
1–3	81	46% [39–53%]	43% [33–55%]	43% [30–56%]	51% [39–65%]
4–6	37	20% [16–28%]	22% [13–31%]	23% [14–36%]	19% [11–32%]
>6	28	16% [11–22%]	17% [10–27%]	24% [14–36%]	8% [3–19%]
Number of psychiatric comorbidities					
0	44	25% [19–32%]	25% [16–36%]	17% [9–30%]	32% [21–46%]
1–3	104	59% [51–66%]	56% [45–67%]	58% [44–70%]	63% [50–75%]
>3^b^	29	17% [12–23%]	19% [12–30%]	26% [15–38%]	4% [0–14%]
Continuous (ordinal)		Median (IQR), [95% CI]			
Age (years)	178	43 (29–53), [39–46%]	44 (29–53), [35–49%]	45 (34–56), [41–52%]	39 (29–49), [33–46%]
Number of past episodes	168	2 (1–5), [2–3%]	2 (1–3), [0–2%]	3 (1–10), [2–5%]	2 (1–5), [1–4%]
Current episode duration (years)^g^	176	5 (1–11), [4–6%]	6 (3–12), [5–10%]	6(1–13), [2–7%]	2(1–9), [2–5%]
MSM	175	8 (7–9), [7–8%]	8 (7–9), [7–8%]	8 (7–9), [7–9%]	8 (7–8), [7–8%]
WSAS total score	177	27 (22–33), [26–30%]	27 (22–32), [24–31%]	29 (21–34), [25–32%]	28 (23–32), [24–30%]
MADRS total score	177	31 (26–36), [29–32%]	28 (25–34), [26–29%]	33 (29–37), [30–35%]	32 (28–36), [29–34%]

Missing data were limited; the only variable with >5% missing data was the number of past episodes (ten missing, notably seven from Oxford and three from London). Between-group differences are detailed in the footnotes (where confidence intervals did not overlap between groups). BAME, Black and minority ethnic; IQR, interquartile range; MSM, Maudsley Staging Model; WSAS, Work and Social Adjustment Scale; MADRS, Montgomery–Åsberg Depression Rating Scale.

a. Newcastle had fewer BAME participants and more White participants than London.

b. Oxford had a higher proportion of employed participants than Newcastle, and fewer participants with more than three psychiatric comorbidities.

c. Oxford and Newcastle participants were significantly less likely than London participants to be recruited from the community.

d. Oxford participants were significantly less likely than Newcastle and London participants to be recruited from primary care.

e. Oxford participants were significantly more likely than Newcastle participants, and both more likely than London participants, to be recruited from secondary care.

f. Concomitant medications (psychotropic and non-psychotropic), not including the number of current antidepressant medications, which is computed elsewhere.

g. Oxford participants had significantly shorter episode durations than London participants.

### Extent of adherence to best-practice care pathways

Table 2 contains the treatment gap outcomes, alongside the best-practice guideline advice for each.

**Table 2:** Treatment gap adherence indications

Variable	*n*	Guideline recommendation	All (*N* = 178)	London (*n* = 73)	Newcastle (*n* = 52)	Oxford (*n* = 53)
			% [95% CI]			
Binary						
Psychotherapy						
Any: yes (versus no)	178	NICE: offer to all (step 3 to all with non-response to step 2)	68% [61–74%]	70% [59–79%]	64% [50–75%]	70% [56–81%]
Adequate: yes (versus no)	177		53% [46–60%]	55% [44–66%]	54% [41–67%]	49% [36–62%]
Adjunctive medication						
Yes (versus no)	175	BAP: after non-response to two ADMs, try combination	38% [31–46%]	25% [17–37%]	54% [41–68%]	40% [28–53%]
Secondary care access						
Yes (versus no)^c^	178	BAP: refer if non-response after two or more ADMs, suicide risk or if GP requires support	44% [37–52%]	23% [15–34%]	46% [33–60%]	72% [58–82%]
Multi-categorical						
Episode onset to first ADM						
Already on treatment	25	BAP: all treated within <3 months of onset (ADM if moderate, severe or chronic MDD)	14% [6–15%]	11% [7–26%]	12% [5–25%]	21% [14–40%]
0–3 months	20		11% [4–13%]	16% [12–33%]	0%	15% [9–32%]
4–12 months	22		13% [5–14%]	13% [8–28%]	14% [7–27%]	12% [6–27%]
13–24 months	19		11% [4–13%]	11% [7–26%]	15% [8–29%]	6% [2–19%]
>24 months	64		36% [18–31%]	28% [24–48%]	54% [43–70%]	29% [24–51%]
Number of psychological therapies						
Any						
0	57	NICE recommends to offer psychological therapy to all	32% [26–39%]	30% [21–41%]	37% [25–50%]	30% [19–44%]
1	61		34% [28–42%]	30% [21–41%]	40% [28–54%]	34% [23–47%]
2	36		20% [15–27%]	23% [15–34%]	14% [6–26%]	23% [13–36%]
3	14		8% [4–13%]	10% [4–19%]	8% [3–19%]	6% [1–16%]
>3	19		6% [3–10%]	6% [3–15%]	2% [0–11%]	8% [2–18%]
Adequate therapy^a^						
0	83		47% [39–54%]	45% [34–57%]	46% [33–60%]	51% [38–64%]
1	66		37% [30–44%]	36% [26–47%]	44% [32–58%]	34% [23–47%]
2	19		11% [7–16%]	15% [8–25%]	6% [1–16%]	9% [4–21%]
3	7		4% [2–8%]	3% [0–10%]	4% [0–14%]	6% [1–16%]
>3	1		1% [0–3%]	1% [0–8%]	0%	0%
Number of antidepressant medications						
Any						
2	72	BAP: after no response to two or more ADMs, try combination or adjunctive treatment NICE: after non-response to ADM or psychological therapy, consider combination	40% [34–48%]	43% [32–55%]	35% [23–48%]	43% [31–57%]
3	42		24% [18–31%]	21% [13–32%]	27% [17–40%]	25% [15–38%]
4	32		18% [13–24%]	21% [13–32%]	12% [5–23%]	21% [12–34%]
5	18		10% [6–16%]	10% [5–19%]	15% [8–28%]	6% [1–16%]
>5	13		7% [4–12%]	6% [2–14%]	11% [5–23%]	6% [1–16%]
Adequate						
2	81		46% [39–53%]	51% [39–62%]	33% [21–46%]	51% [40–66%]
3	37		21% [16–28%]	18% [11–28%]	25% [15–38%]	21% [12–35%]
4	33		19% [14–25%]	19% [12–30%]	19% [11–32%]	17% [9–30%]
5	16		9% [6–14%]	8% [4–17%]	15% [8–28%]	4% [0–14%]
>5	9		5% [3–10%]	4% [1–12%]	7% [3–19%]	2% [0–14%]
First ADM duration						
0–6 weeks	12	NICE/BAP: remain on ADM for 4–8 weeks before switching if non-response after dose increase	7% [4–12%]	8% [4–18%]	4% [0–14%]	8% [3–19%]
7–16 weeks^b^	77		43% [38–52%]	26% [19–40%]	69% [56–80%]	42% [30–56%]
17–52 weeks	41		23% [18–31%]	29% [21–43%]	12% [5–23%]	26% [17–40%]
>52 weeks	42		24% [19–31%]	30% [22–44%]	15% [8–29%]	23% [14–36%]
Second ADM duration						
0–6 weeks	15	NICE/BAP: remain on ADM for 4–8 weeks before switching if non-response after dose increase	8% [5–14%]	11% [8–30%]	0%	13% [6–26%]
7–16 weeks^d^	64		36% [31–45%]	16% [15–39%]	71% [60–84%]	28% [18–42%]
17–52 weeks	43		24% [19–32%]	33% [25–47%]	12% [5–24%]	25% [15–38%]
>52 weeks	48		27% [22–35%]	33% [25–47%]	14% [7–27%]	32% [21–46%]
Number of first- or second-line adjunct medications						
Any^b^						
0	158	BAP: after non-response to two or more ADMs, try combination or adjunctive treatment	89% [84–93%]	64: 88% [78–94%]	47: 90% [81–97%]	47: 89% [77–95%]
1	15		8% [5–14%]	7: 10% [4–19%]	2: 4% [0–14%]	6: 11% [5–23%]
2	3		2% [0–5%]	2: 3% [0–10%]	1: 2% [0–11%]	0: 0%
3	1		1% [0–3%]	0: 0%	1: 2% [0–11%]	0: 0%
Adequate						
0	161		90% [88–96%]	90% [81–96%]	90% [93–100%]	91% [86–100%]
1^d^	10		6% [3–10%]	7% [3–15%]	0%	9% [4–22%]
2	2		1% [0–4%]	3% [0–10%]	0%	0%

In the table where totals do not add to 100, this is attributable to missing data points. Missing data limited; the only variable with >5% missing data was the delay to first treatment within episode (28 missing; 16 from London, 3 from Newcastle and 9 from Oxford). Between-group differences are detailed in the footnotes (where confidence intervals did not overlap between groups). MDD, major depressive disorder; NICE, National Institute for Health and Care Excellence; BAP, British Association for Psychopharmacology; ADM, antidepressant medication; GP, general practitioner.

a. Adequacy of therapy was defined according to NICE guidelines (pertaining to low-intensity interventions) in terms of modality and intensity e.g. equivalent of a minimum of six sessions of individual cognitive–behavioural therapy, or ten sessions of group cognitive–behavioural therapy.

b. More participants from Newcastle than London had been treated with adjunct pharmacotherapy. Additionally, more than been treated with their first antidepressant medication for between 7 and 16 weeks.

c. More participants from Oxford than London had accessed secondary care.

d. Fewer participants from Newcastle (0) than other sites had spent <7 weeks taking their second antidepressant medication, but more spent between 7 and 16 weeks taking this medication. Also, fewer participants from Newcastle than other sites had been treated with one adequate first- or second-line augmentation agent.

#### Time to antidepressant initiation

Contrary to guidelines, the most common duration between episode onset and antidepressant treatment was >24 months (assessed as a categorical variable; 36%; 95% CI 18–31%), with a combined total of 60% of the sample not receiving treatment within 3 months. This factor differed between site, with no participants from Newcastle initiating treatment before 4 months after their episode onset, discounting those already on treatment (e.g. those who had relapsed when taking an antidepressant). Only 12% of Newcastle participants were pharmacologically treated by 1 year after episode onset, and 54% waited over 2 years to receive medication. In contrast, 36% of participants from Oxford and 27% from London were already on treatment before episode onset, or had initiated treatment within 3 months.

#### Access to psychological therapies

Stepped care recommends that psychological therapies are offered in all stages, including step 2 and step 3. A total of 68% of participants reported receiving some psychological therapy during their current depressive episode, and 53% had completed a therapy course that may be considered adequate. Including all participants, an average of 1.3 (95% CI 1.1–1.5) therapies (any), or 0.7 (95% CI 0.6–0.9) adequate therapies had been undertaken. Considering both pharmacological and psychological therapies together, the proportion of participants who had not received an adequate treatment of either within the first 2 years of their episode was 20.3%.

#### Time to medication changes

The largest proportion of participants (43%) took their first antidepressant for 7–16 weeks (95% CI 38–52), and 36% reported the same timeframe for their second antidepressant (95% CI 31–45). Overall, around half remained on an unsuccessful antidepressant trial for >16 weeks (47% for the first antidepressant, 51% for the second antidepressant), including 24% who remained on their first antidepressant, and 27% on their second antidepressant for over a year.

#### Steps to adjunctive treatment

A total of 55% of participants had undertaken at least three courses of different antidepressants (of which 21% had three, 19% had four, 9% had five and 5% had six or more). Only 38% of the sample had been treated with adjunctive pharmacotherapy in some form. Further, 11% had been prescribed between one and three recommended adjunctive medications, and 7% had received at least one adequate trial.

#### Access to secondary care

A total of 44% of the full sample currently had a psychiatrist (95% CI 37–52), but this rate was much lower in London than the other sites (23% in London, 46% in Newcastle, 72% in Oxford). It is notable that many participants were actively recruited from secondary care services in Oxford (87%) and Newcastle (66%), which may have influenced these differences.

#### Requirement for step 4 intervention

One participant in this sample had received ECT in their current episode. This individual had been on two antidepressants, with no history of psychological therapies or adjunctive treatments in their episode, without access to secondary care. Two participants had been admitted as in-patients; these participants and three others had attended an accident and emergency department for reasons related to mood disorder (e.g. self-harm) in the past 3 months. This outcome is not included in tables because of the low incidence of step 4 interventions.

### Associations between treatment gaps and participant characteristics

Tables 3 and 4 display treatment gap outcomes compared with participant characteristics. Significant associations are summarised below.

**Table 3:** Continuous treatment gap outcomes compared with characteristics

Characteristic	Time to treatment after episode onset	Time on first antidepressant medication	Time on second antidepressant medication	Number of antidepressant medications
Binary: Mann–Whitney *U*-test				
Female versus male	*U* = 2861, *P* = 0.735	*U* = 3764, *P* = 0.748	*U* = 3442, *P* = 0.649	*U* = 3522, *P* = 0.315
Employed versus unemployed	***U*** **=** **3304, *P*** **=** **0.045**^a^	*U* = 3851, *P* = 0.658	*U* = 3850, *P* = 0.401	*U* = 4028, *P* = 0.607
Long-term relationship, yes/no	*U* = 2.491, *P* = 0.228	*U* = 3.093, *P* = 0.213	*U* = 2.025, *P* = 0.363	*U* = 1.816, *P* = 0.403
Physical comorbidity, yes/no	*U* = 2130, *P* = 0.059	*U* = 1.759, *P* = 0.624	*U* = 2.337, *P* = 0.506	*U* = 2334, *P* = 0.852
Lifetime suicide attempt, yes/no	*U* = 2688, *P* = 0.942	*U* = 1.696, *P* = 0.638	*U* = 2.580, *P* = 0.461	*U* = 3976, *P* = 0.331
Ethnicity (White/BAME)	*U* = 1205, *P* = 0.204	*U* = 1581, *P* = 0.054	*U* = 1552, *P* = 0.065	*U* = 1461, *P* = 0.802
Multi-categorical: ANOVA				
Recruitment method (groups)	*F*(3) = 0.558, *P* = 0.644	*F*(3) = 2.334, *P* = 0.076	*F*(3) = 2.351, *P* = 0.074	*F*(3) = 2.464, *P* = 0.064
Education (groups)	*F*(4) = 1.33, *P* = 0.261	*F*(4) = 0.498, *P* = 0.737	*F*(4) = 0.704, *P* = 0.590	*F*(4) = 0.925, *P* = 0.451
Continuous: Spearman's correlation				
Age	*r* = 0.102, *P* = 0.213	*r* = 0.149, *P* = 0.052	*r* = 0.109, *P* = 0.157	*r* = −0.086, *P* = 0.259
Number of current medications	***r*** **=** **0.219, *P*** **=** **0.007**^a^	*r* = 0.028, *P* = 0.717	*r* = 0.138, *P* = 0.073	*r* = 0.106, *P* = 0.162
Number of psychiatric comorbidities	***r*** **=** **0.184,** *P* **=** 0**.025**^a^	*r* = 0.042, *P* = 0.582	*r* = 0.031, *P* = 0.689	*r* = 0.052, *P* = 0.495
Number of episodes	***r*** **=** **−0.378,** *P* **≤** 0**.001**^a^	*r* = 0.087, *P* = 0.268	*r* = **−**0.018, *P* = 0.818	***r*** **=** **−0.191,** *P* **=** **0.014**^b^
Current episode duration	***r*** **=** **0.627, *P*** **< 0.001**^a^	*r* = 0.117, *P* = 0.128	***r*** **=** **0.204, *P*** **=** **0.008**^c^	***r*** **=** **0.298, *P*** **=** **0.000**^b^
MSM	***r*** **=** **0.425, *P*** **<** **0.001**^a^	*r* = **−**0.087, *P* = 0.260	*r* = **−**0.206, *P* = 0.783	***r*** **=** **0.770, *P*** **=** **0.000**^b^
WSAS total score	*r* = 0.053, *P* = 0.523	*r* = **−**0.013, *P* = 0.868	*r* = **−**0.061, *P* = 0.434	*r* = 0.065, *P* = 0.392
MADRS total score	*r* = 0.108, *P* = 0.192	*r* = **−**0.015, *P* = 0.846	*r* = **−**0.007, *P* = 0.931	***r*** **=** **0.155, *P*** **=** **0.040**^b^

Data points in bold reflect statistically significant results at *P* < 0.05, and details around direction of effect are noted in the footnotes. BAME, Black and minority ethnic; MSM, Maudsley Staging Model; WSAS, Work and Social Adjustment Scale; MADRS, Montgomery–Åsberg Depression Rating Scale.

a. Participants with a longer delay to treatment after episode onset were more likely to be unemployed (than employed), be on a greater number of medications, have higher psychiatric comorbidity, a lower number of previous episodes, a longer current episode duration and more severe treatment resistance (MSM score).

b. Participants who had taken more antidepressant medications in their episode had a lower number of previous episodes, a longer current episode duration, more severe treatment resistance (MSM score) and more severe depression symptoms (MADRS).

c. Participants who spent more time on their second antidepressant medication had longer episode durations.

**Table 4:** Categorical treatment gap outcomes compared with characteristics

Characteristic	Access to psychological treatment	Adjunctive treatment	Access to secondary care^a^	
			Non-secondary recruitment (*n* = 89)	Secondary recruitment (*n* = 85)
Categorical (nominal): *χ*^2^-test				
Female versus male	X^2^ = 0.024, *P* = 0.876	X^2^ = 0.2.81, *P* = 0.094	X^2^ = 2.14, *P* = 0.144	X^2^ = 2.45, *P* = 0.620
Employed versus unemployed	X^2^ = 2.643, *P* = 0.104	X^2^ = 3.52, *P* = 0.061	X^2^ = 1.33, *P* = 0.249	X^2^ = 0.049, *P* = 0.825
Long-term relationship, yes/no	X^2^ = 0.750, *P* = 0.386	X^2^ = 0.222, *P* = 0.638	X^2^ = 2.98, *P* = 0.084	X^2^ = 0.665, *P* = 0.415
Physical comorbidity, yes/no	X^2^ = 0.001, *P* = 0.972	X^2^ = 0.283, *P* = 0.595	X^2^ = 0.095, *P* = 0.757	X^2^ = 0.420, *P* = 0.517
Lifetime suicide attempt, yes/no	X^2^ = 0.070, *P* = 0.792	X^2^ = 3.46, *P* = 0.063	X^2^ = 0.014, *P* = 0.905	X^2^ = 0.134, *P* = 0.715
Recruitment method (groups)	X^2^ = 2.288, *P* = 0.319	**X^2^ = 7.34, *P* = 0.025^b^**	**X^2^ = 30.14, *P* < 0.001^c^**	
Ethnicity (groups)	X^2^ = 1.094, *P* = 0.579	X^2^ = 0.163, *P* = 0.922	X^2^ = 3.15, *P* = 0.206	X^2^ = 0.224, *P* = 0.894
Education (groups)	X^2^ = 0.828, *P* = 0.935	X^2^ = 1.45, *P* = 0.836	X^2^ = 2.8, *P* = 0.592	X^2^ = 1.55, *P* = 0.818
Continuous (ordinal): Mann-Whitney *U*-test				
Age	*U* = 3401, *P* = 0.141	*U* = 4070, *P* = .166	***U* = 367, *P* = 0.003^c^**	*U* = 1084, *P* = 0.062
Number of current medications	*U* = 3707, *P* = 0.661	*U* = 4120, *P* = 0.094	*U* = 648, *P* = 0.939	*U* = 1063, *P* = 0.061
Number of psychiatric comorbidities	***U* = 3721, *P* = 0.688**	*U* = 3407, *P* = 0.576	*U* = 760, *P* = 0.276	*U* = 789, *P* = 0.521
Number of episodes	*U* = 2735, *P* = 0.012^d^	*U* = 3171, *P* = 0.786	*U* = 465, *P* = 0.232	*U* = 617, *P* = 0.150
Current episode duration	*U* = 4508, *P* = 0.052	*U* = 3513, *P* = 0.824	*U* = 599, *P* = 0.567	*U* = 1038, *P* = 0.106
MSM	***U* = 4452, *P* = 0.033^d^**	*U* = 3746, *P* = 0.423	*U* = 641, *P* = 0.878	*U* = 1001, *P* = 0.094
WSAS total score	*U* = 3637, *P* = 0.520	*U* = 3587, *P* = 0.943	*U* = 729, *P* = 0.459	*U* = 1028, *P* = 0.103
MADRS total score	*U* = 3271, *P* = 0.083	*U* = 4091, *P* = 0.102	*U* = 687, *P* = 0.749	***U* = 1185, *P* = 0.003^c^**

Data points in bold reflect statistically significant results at *P* < 0.05 and details around direction of effect are noted in the footnotes. MSM, Maudsley Staging Model; WSAS, Work and Social Adjustment Scale; MADRS, Montgomery–Åsberg Depression Rating Scale.

a. Because access to secondary care is so substantially influenced by recruitment method (community versus primary care versus secondary care), these comparisons were undertaken separately for participants recruited via secondary care (*n* = 65 for those recruited through secondary care with current secondary care access, and *n* = 22 for those without current access; *n* = 14 for those recruited either through primary care or the community with current secondary care access, and *n* = 76 for those without current access). Anecdotally, the primary reasons for the individuals having been recruited via secondary care but having no ongoing access to secondary care are perceived to be because of discharge or new referral without full access to the service by the time of the Lithium versus Quetiapine in Depression study baseline assessment.

b. Those recruited from secondary care were more likely to have received adjunctive pharmacological treatment than those recruited from primary care and the community.

c. Younger participants (not recruited in secondary care) were more likely to have access to a psychiatrist than older participants, and those with more severe depression (recruited through secondary care) were more likely to be under the care of a secondary care mental health clinician.

d. Those who had undertaken at least one adequate psychological therapy had a lower number of episodes and more severe treatment resistance.

#### Time to antidepressant initiation

Those with a longer duration between episode onset and treatment were more likely to be unemployed (*U* = 3304, *P* = 0.045), be on more concomitant medications (*r* = 2.19, *P* = 0.007), have a higher number of psychiatric comorbidities (*r* = 1.84, *P* = 0.025), have a lower number of previous episodes (*r* = −0.241, *P* = 0.004), have a longer current episode (*r* = 0.381, *P* < 0.001) and have more severe treatment resistance (*r* = 0.179, *P* = 0.027).

#### Time to medication changes

Treatment length for the second antidepressant prescribed was associated with a longer duration of episode (*r* = 0.204, *P* = 0.008).

#### Steps to adjunctive treatment

Participants who had tried more antidepressants in the current episode had fewer previous depressive episodes (*r* = −0.191, *P* = 0.014), but a longer current episode duration (*r* = 0.298, *P* < 0.001), more severe treatment resistance (*r* = 0.770, *P* < 0.001) and more severe depressive symptoms at present (*r* = 0.155, *P* = 0.040). The latter three findings are interrelated, since the measure of treatment resistance used (the MSM) includes factors assessing episode duration, severity of symptoms and number of antidepressants taken in the current episode. Those who had tried more antidepressants in their current episode also experienced longer delays to initial treatment (*r* = 0.224, *P* = 0.006).

#### Access to psychological therapy

Those who had completed at least one adequate psychological therapy had fewer previous episodes (*U* = 2735, *P* = 0.012) and more severe treatment resistance (*U* = 4452, *P* = 0.033).

#### Access to secondary care

Participants who were recruited from secondary care were more likely to have received adjunctive pharmacological treatment than those recruited from primary care and the community (*χ*^2^ = 6.66, *P* = 0.017). Of those not recruited directly from secondary care, younger participants were more likely to have access to a psychiatrist than older participants (*U* = 367, *P* = 0.003)**.** Among those recruited via secondary care (but not necessarily still attending secondary care; see Table 2), those with more severe depression were more likely to retain access to a psychiatrist (*U* = 1185, *P* = 0.003).

## Discussion

This UK sample of 178 participants suffering from TRD frequently experienced treatment gaps with regards to the recommendations made in the NICE stepped care pathway. This includes long delays to treatment within their current episode, with only approximately a quarter receiving antidepressants within the first 3 months, a further 13% waiting up to 1 year and 36% waiting more than 2 years after episode onset. Although two-thirds of the sample had received a psychological therapy, only half of participants had undertaken a therapy in accordance with treatment guidelines (in terms of therapeutic modality and intensity), with 16% having undergone more than one adequate psychological therapy. Despite almost half of the sample having accessed secondary care services and a third having received some form of adjunctive medication, only 11% were treated with a recommended first- or second-line adjunctive agent and only 7% received an adequate course. Moreover, 21% had received three, 19% had four and 14% had five or more courses of antidepressant monotherapy in the current episode. Despite their lack of efficacy, half of first and second step antidepressant treatments were continued for 17 weeks or longer, and a quarter were continued for over a year.

Although not all patients may desire or be able to engage with a guideline-approved psychological therapy, the finding that only approximately half had completed an adequate course of therapy represents a divergence from treatment guidelines.^6^ Our results suggest that this treatment gap may be particularly notable for those who had fewer depressive episodes but more severe treatment resistance within the current episode. The NHS Improving Access to Psychological Therapies (IAPT) service is the main point of access to psychological therapies for those with depression in the UK, but timely access to IAPT services is a critical issue, with one in ten patients waiting over a year to receive treatment.^27^ Another barrier to therapy could include more severe illness (which has been associated with difficulties in both initial access and subsequent engagement with psychological therapies^28^). It is important to note that completion of, or response to, psychotherapy does not contribute to commonly used definitions of TRD.^29^

We report a long delay before treatment switch and a high number of antidepressants taken in the current episode, contrary to current NICE guidelines, which recommend a 4- to 8-week course of antidepressants before switching if ineffective, and two failed treatments before a trial of an adjunctive intervention. Such delays are likely to contribute to an increased burden of illness in these patients, which might be readily avoided by adhering more closely to treatment guidelines and following a measurement-based care programme.^6,11^ Most patients had not tried any adjunctive pharmacotherapy treatment when joining the study, which may not appear unexpected, since it is only after two ineffective antidepressant treatments for depression that patients should be considered for first-line adjunctive treatment. It is worth noting that study eligibility criteria excluded any individuals who had been initiated on adequate doses of lithium or quetiapine (two of the first-line augmenting agents) in the current episode. Therefore, the LQD sample may have selectively recruited a sample not treated in line with guidance for this step. Having said that, many other first- and second-line adjunctive treatments are commonly used in TRD,^14,23^ and even allowing for the exclusion of lithium and quetiapine, given the current episode duration and number of treatments trialled within the episode, a higher rate of adjunctive treatment would have been expected in these patients.

Participants were predominantly recruited from secondary care at the Oxford and Newcastle sites, but this rate was much lower for London participants. Guidelines recommend access to psychiatric services before considering adjunctive treatment, so the subset of London LQD participants might be considered more representative of those living with TRD in the community. By design, the study is likely to identify a disproportionate number of patients with TRD in primary care who have not received adjunctive treatment, excluding those who had already received lithium or quetiapine; although from reviewing records of individuals screened and excluded, :1% of those otherwise eligible were excluded on this basis. We therefore speculate that this has not markedly affected the representativeness of our sample.

Some participants were recruited via secondary care but did not have ongoing care from a psychiatrist when entering the LQD study. This could be because they had been discharged by the time they entered the LQD study or because patients were being recruited at the point of entry into the secondary care service. This is most likely the case for Oxford participants, as participants were recruited into the study upon referral to the specialist TRD service. Therefore, these participants were not under ongoing secondary care at the point of entering the study. We are not aware of any previous data addressing the rate of secondary care access in individuals with TRD in the UK as a whole. There may also be a disparity of access between different areas of England, as (despite alignment of recruitment strategies between sites) markedly fewer participants could be recruited from secondary care services in London than the other sites, although this might also reflect differences in recruitment infrastructures at each site. Nevertheless, the suggestion is that overall (and especially in areas like London), a large number of patients with TRD are being managed solely in primary care despite recommendations to the contrary, and to the likely detriment of available treatment options and longer-term health outcomes.

With a median episode duration of 5 years, a considerable proportion of this sample may meet criteria for dysthymia. We found that longer episodes were associated with longer delays to initial treatment, more time spent before switching medications and a greater number of antidepressants trialled. This may support a relationship between dysthymia and treatment resistance,^30^ and consolidates the importance of adequately treating this group in the early stages of their condition.^31^

It is noteworthy that 36% of the sample's current episodes were not treated pharmacologically for 2 years or more following the onset of a depressive episode. Around half the cohort did receive an adequate course of psychological therapy at some point within the 2-year period. Therefore, a significant proportion of our TRD sample (20%) were not adequately treated with medication or psychotherapy for 2 or more years from episode onset. Our results further indicate that this subset experienced more severe treatment resistance, a greater number of psychiatric comorbidities and a longer current episode. In Germany, Trautmann and Beesdo-Baum^8^ also found that around 30% of patients with depression were not allocated psychotherapy or antidepressants by primary care physicians, with the most pronounced signs of undertreatment being found in severe depression. The relationship between symptom severity and undertreatment in depression has also been found in the USA.^32^ In addition, those who experienced longer delays before receiving their initial antidepressant treatment in the index episode had also subsequently been treated with more antidepressant medications in that episode. This finding is consistent with evidence showing that longer delays to treatment for depression are associated with a higher severity of treatment resistance.^16^ This represents a key opportunity for care pathway optimisation, to reduce the burden and prevalence of treatment resistance and improve outcomes.

Individuals experiencing longer delays before receiving adequate, guideline-recommended treatment appear to be a more complex group that bear a greater illness burden, and it may be that depression treatment had previously been overlooked because of the prioritisation of other physical and psychiatric conditions, or underdetection of their depression. However, there may be other factors playing a role, such as a patient's willingness, cooperation, capacity and insight into their own condition. This group could also include individuals with longstanding psychosocial or personality difficulties. Additionally, the treatment gaps identified may represent failings in the stepped care pathway itself, to better reflect and incorporate patient preference and the capacity of secondary care services. Non-adherence to guidelines could be a result of a multitude of patient- and/or practitioner-related factors that we were unable to control for in this study.

Despite a general consensus in the utility of stepped care models for depression, there is a lack of standardisation across treatment guidelines in several domains, including the sequence and number of steps, the treatment components incorporated and the assessment of when it is appropriate to ‘step up’, which appears to be largely down to individual clinical judgement.^33,34^ Although it is important to have a level of flexibility within the model to allow for individual differences, heterogeneity across guidelines leads to discrepancies in the application of stepped care systems, uncertainty in the definition of best practice and could exacerbate the emergence of treatment gaps. It is also possible that these guidelines do not sufficiently account for resource constraints in healthcare systems. Pending the emergence of further evidence, greater collaborations between healthcare providers (including resource allocators) within guideline-development committees could help add to guidelines and minimise treatment gaps.

The main limitation of this study is that we were unable to design our measures fully in line with NICE guideline recommendations, as this was not the primary aim of the LQD study. Consequently, we were limited in the depth of our investigation and unable to look at several guideline components, including date of first contact with services, active monitoring by primary care clinicians and frequency of contacts with secondary health clinicians. We were also limited to investigating these variables within a patient's current episode; it would have been of additional interest to look at the lifetime care pathways for this population and explore the initial treatment of this sample, comparing divergence from guidelines between lifetime and current episode. Additionally, we were limited to a relatively small sample of research participants who may not have been representative of the TRD population. For example, their preference may be more aligned with pharmacotherapies than psychological therapies, notwithstanding a relatively high rate of previous psychological therapy.

We emphasise that this is a first step that does not yet provide information on the effects of moving toward greater adherence to best-practice guidelines. We also have not been able to fully elucidate the reasons for treatment gaps, which could include a range of factors related to the patient or at a physician and service level. Future research exploring outcomes based on differing levels of adherence to best-practice guidelines would provide greater insight.

Another limitation is the retrospective nature of some measures, necessitating reliance on participants’ memory for a number of variables, including episode onset, delays to initial treatment and treatment duration for each antidepressant (although this is reflective of clinical care in general, which is partially dependent on patient recall of illness characteristics and timelines). Finally, although our sample includes data from three different regions of the UK, our findings may not be representative of the entire country. We have already indicated large disparities between care pathways in Newcastle, Oxford and London. Although these differences could be attributed to inter-site rather than regional variations, there may be significant regional heterogeneity in access to different services and different care pathways in place.

A longitudinal naturalistic study, without the eligibility criteria of a randomised controlled trial, would capture a greater range of individual trajectories and generate a more complete picture of the current care of this population. Future research could also explore these pathways in a more widespread context, across regions of the UK and internationally.

To our knowledge, this is the first study to explore the treatment pathways in a sample of participants with established TRD, allowing for the assessment of variable access to different care pathways and direct comparisons between the recommended guidelines and the reality of care for people with TRD in the UK. Our findings demonstrate some clear treatment gaps between best-practice guidelines and the reality of depression treatment for those with TRD in the UK, as well as clear variation across the country. There is a need for improvement in models of care to ensure that the care of patients with MDD – and particularly those with TRD, who incur the most illness burden – can be optimised to ultimately enhance short- and long-term health outcomes.

## Data Availability

The data are not publicly available due to ethical approval restrictions. Please contact A.J.C. or the corresponding author for data availability requests.
